# Airway Colonization in Children with Medical Complexity: Challenges and Management Strategies

**DOI:** 10.3390/jcm14030848

**Published:** 2025-01-27

**Authors:** Valentina Fainardi, Luisa Rizzo, Giulia Zambelli, Martina Berzieri, Erika Maugeri, Elena Giacalone, Roberta Carbone, Maria Carmela Pera, Susanna Esposito

**Affiliations:** Pediatric Clinic, Department of Medicine and Surgery, University of Parma, 43126 Parma, Italy; valentina.fainardi@unipr.it (V.F.); luisa.rizzo@unipr.it (L.R.); giulia.zambelli@unipr.it (G.Z.); martina.berzieri@unipr.it (M.B.); erika.maugeri@unipr.it (E.M.); elena.giacalone@unipr.it (E.G.); roberta.carbone2@unipr.it (R.C.); mariacarmela.pera@unipr.it (M.C.P.)

**Keywords:** airway clearance therapy, airway colonization, antibiotics, medical complexity, respiratory infections

## Abstract

Over recent years, advances in medical care have significantly improved the survival of children with severe chronic conditions. These children, referred to as children with medical complexity (CMC), present unique and demanding healthcare challenges. Although definitions of CMC remain inconsistent, these patients are typically characterized by chronic, often severe conditions requiring daily specialized treatments and the use of various medical devices. CMC represent a substantial burden for healthcare systems due to their high medical costs, and place considerable strain on caregivers, who must provide continuous assistance. Airway colonization by pathogens such as *Pseudomonas aeruginosa*, methicillin-resistant *Staphylococcus aureus* (MRSA), and *Haemophilus influenzae* is common in CMC and contributes to recurrent respiratory infections, increased hospitalizations, and progressive lung damage. The management of airway colonization in this population is a topic of ongoing debate, often involving a combination of airway clearance techniques (ACT) and antibiotic therapies. Antibiotics may be administered systemically, nebulized, or in combination, depending on the clinical context and severity of the condition. This review highlights the complexities of managing airway colonization in CMC, emphasizing the need for tailored therapeutic approaches to mitigate respiratory complications and improve outcomes.

## 1. Introduction

Over the last decades, advances in science, technology, and neonatal and pediatric intensive care have dramatically improved the survival rates of children with congenital diseases, those in critical conditions following acute events, oncological diseases, or extreme prematurity [1,2]. Among these, children with pronounced healthcare utilization, functional limitations, and significant family needs are categorized as children with medical complexity (CMC) [1,2,3,4,5]. These children often endure severe, lifelong, and incurable health conditions. While some of their health challenges are inherently complex due to rarity and pathophysiology, others result from clinical interactions of multiple conditions, which impact their health status and quality of life. Managing these complications is particularly challenging due to the need for extensive coordination across multiple providers [6,7].

CMC typically require care from multidisciplinary teams that include pediatricians, pulmonologists, neurologists, gastroenterologists, nutritionists, respiratory physiotherapists, and specialized nurses managing devices such as tracheostomies and gastrostomies [8,9]. Though accounting for less than 1% of the pediatric population, CMC represent a disproportionate social and economic burden, contributing to 33% of pediatric healthcare expenditures, 37% of hospitalizations, and nearly 60% of hospital costs [7].

A comprehensive understanding and identification of CMC is essential to optimize care and support for patients and families [8]. The most widely accepted definition of CMC, introduced by Cohen in 2011, identifies four domains: diagnostic conditions, functional limitations, family needs, and increased healthcare utilization [7,9]. However, this definition lacks detailed operationalization of these domains. More recent frameworks, including the pediatric medical complexity algorithm (PMCA) and a Delphi consensus study, have attempted to standardize these criteria and provide robust classification systems (Supplementary Materials S1) [1,3,10,11,12,13,14,15,16,17,18,19,20,21]. The definition provided by the American Academy of Pediatrics through the modified Delphi study by Millar et al. characterizes CMC using four key domains [3]. These include chronic, often severe medical conditions that are typically lifelong or incurable, such as genetic syndromes, neurological impairments, or organ system failures. Additionally, CMC experience significant functional limitations that impair physical, developmental, or cognitive functioning, often requiring assistive devices or technologies. The definition also highlights the elevated caregiving demands placed on families, including the need for specialized training, coordination of care, and emotional and financial support to manage the child’s condition effectively. Finally, these children have frequent and complex interactions with healthcare systems, involving regular specialist visits, hospital admissions, medical equipment use, and dependency on advanced medical therapies. This comprehensive framework provides a standardized approach to identifying and addressing the unique needs of CMC, facilitating improved clinical care, research, and policy development [3].

Airway colonization, a critical concern for CMC, has received limited attention despite its association with recurrent respiratory infections, progressive lung damage, and the development of multidrug-resistant organisms. This narrative review explores the prevalence, risk factors, and therapeutic strategies for airway colonization in this vulnerable population, highlighting priorities for future research. By consolidating knowledge on this topic, this work intends to inform clinicians and researchers about the respiratory challenges faced by CMC and guide future research and therapeutic approaches. This manuscript focuses on CMC as a broader group due to their unique and varied healthcare challenges that often involve multiple organ systems and require interdisciplinary care. Although conditions like cystic fibrosis (CF) and non-CF bronchiectasis represent significant causes of chronic respiratory infection in children, they are distinct entities with well-established management frameworks and a narrower scope compared to the multifaceted needs of CMC. Thus, this review prioritizes the wider spectrum of respiratory complications and care strategies relevant to the heterogeneous population of CMC.

This narrative review was conducted by systematically searching peer-reviewed literature in major medical databases, including PubMed, Scopus, and Web of Science, using the following keywords: “children with medical complexity”, “airway colonization”, “respiratory infections”, “tracheostomy”, and “multidrug-resistant organisms”. Relevant studies published in English from 2000 to 2024 were considered. Articles were selected based on predefined inclusion and exclusion criteria. Inclusion criteria included studies that focused on airway colonization and respiratory infections in CMC, including cohort studies, randomized controlled trials, case-control studies, and well-documented case series. Eligible studies provided data on epidemiological aspects, risk factors, clinical management, or therapeutic interventions, such as airway clearance techniques, antibiotic therapies (systemic or nebulized), or the role of devices like tracheostomies. Exclusion criteria were studies that lacked sufficient detail on airway colonization or respiratory infections in the CMC population, reviews without primary data, studies focusing exclusively on conditions like cystic fibrosis or non-CF bronchiectasis, or research limited to adult populations. Additionally, the references within selected articles were screened to identify further relevant studies. This rigorous selection process aimed to ensure a comprehensive and focused analysis of the key issues related to airway colonization and respiratory infections in this vulnerable population.

## 2. Respiratory System in Children with Medical Complexity (CMC)

The respiratory system is frequently affected in CMC, often leading to recurrent respiratory infections and airway colonization by resistant pathogens [4,19]. Several factors contribute to these challenges. For instance, the weakness of respiratory muscles, commonly observed in children with cerebral palsy or neuromuscular diseases, reduces the effectiveness of breathing, leading to hypoventilation and difficulties in clearing mucus from the airways [4,19]. Chronic hypoventilation and mucus retention can result in hypercapnia due to carbon dioxide (CO_2_) retention, reduced vital capacity, chronic atelectasis, and recurrent pulmonary infections. Impaired cough reflexes and immobility, which are often observed in this population, further contribute to mucus persistence and subsequent colonization by pathogenic strains [4,19].

Chronic bed rest and hypomobility, typical among CMC, contribute to musculoskeletal complications, such as scoliosis [22]. Scoliosis can develop as a result of muscle weakness, particularly in individuals with neuromuscular disorders, where insufficient muscle strength and control fail to provide adequate support for spinal alignment. These deformities reduce lung capacity and impair ventilation, particularly affecting the lower parts of the lungs, where mucus stagnation and bacterial proliferation are facilitated [22]. Over time, these changes frequently lead to restrictive lung disease and progression to respiratory failure in many patients.

Comprehensive respiratory assessment is crucial for the effective management of these patients [1,2]. Such assessment should include clinical examination, measurement of oxygen saturation and end-tidal CO_2_, regular testing of airway pathogens (using swabs or aspirates), lung function tests, and peak cough flow (PCF) measurements when feasible. Furthermore, as many CMC are affected by neurologic, musculoskeletal, and craniofacial abnormalities, polysomnography is often necessary to detect sleep-disordered breathing, such as hypoventilation or central and obstructive apneas [23,24]. These findings can guide the initiation of non-invasive ventilation.

Physiotherapists experienced in airway clearance techniques play a vital role in managing respiratory secretions [25]. Their support is essential to reduce infections, improve ventilation, and enhance the quality of life for these children. A multidisciplinary approach that addresses the specific respiratory challenges of CMC is indispensable for improving outcomes in this vulnerable population.

## 3. Pulmonary Aspiration

Children with developmental disorders frequently experience dysphagia, characterized by incoordination between swallowing and breathing, and often lack the protective cough reflex needed to prevent food or liquid from entering the larynx or subglottic space [26]. This condition significantly increases the risk of pulmonary aspiration [27]. Aspiration may be evident during meals, presenting as respiratory distress, or it can occur silently. Typical clinical signs include wet voice, wet-sounding breathing, stridor, gagging, tachypnea, or apnea episodes during feeding. In the absence of overt coughing while eating or drinking, additional findings such as persistent chest rattling, intermittent fever, failure to thrive, and recurrent respiratory infections may indicate aspiration. These symptoms warrant an evaluation by a speech and language pathologist to assess their swallowing function. The gold standard for diagnosing aspiration remains the video fluoroscopic swallow study (VFSS) or fibreoptic endoscopic evaluation of swallowing (FEES) [28]. Both techniques offer critical insights into swallowing mechanics and the risk of aspiration.

Another common cause of pulmonary aspiration in children with CMC is gastroesophageal reflux, often associated with gastrointestinal dysmotility in this population [29].

Conservative management strategies for aspiration include the use of food thickeners, specific positioning techniques, and specialized feeding tools [30]. For children with severe dysphagia, poor nutritional status, or frequent respiratory infections, gastrostomy may be necessary [30]. Optimal treatment decisions require an open and collaborative dialogue with the family, carefully weighing the benefits and risks to achieve the best possible outcome for the child. Tailored interventions and ongoing multidisciplinary support are key to managing aspiration effectively and mitigating its impact on respiratory health.

## 4. Airway Colonization in Children with Medical Complexity (CMC)

**Figure 1:**
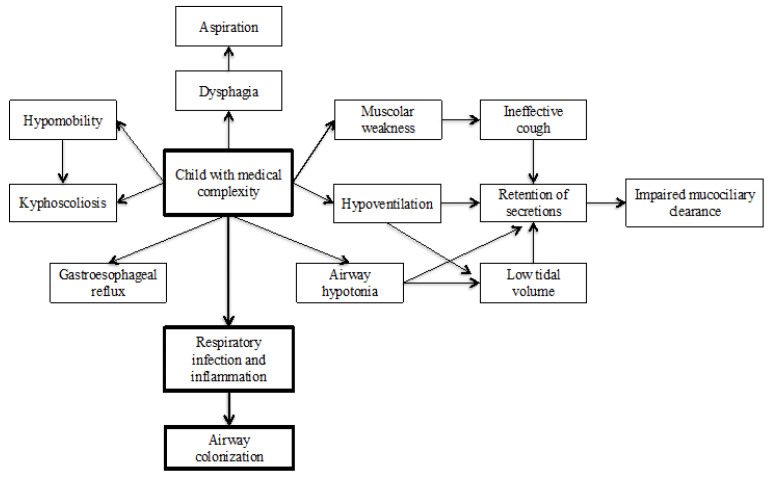
Main factors contributing to respiratory infections and airway colonization of children with medical complexities (CMC).

In CMC, epidemiological data on airway colonization are limited. Frequent use of antimicrobial agents, mucus retention, and chronic inflammation predisposes these children to alterations in airway microbiota, allowing persistent colonization by certain bacterial species, which may exacerbate disease severity [31]. Additionally, life-saving devices like tracheostomy, gastrostomy, and mechanical ventilators significantly increase the risk of contamination and colonization with resistant pathogens. The most commonly implicated organisms are *Pseudomonas aeruginosa* and *Staphylococcus aureus*, including methicillin-resistant *Staphylococcus aureus* (MRSA) [32]. Figure 1 highlights the main factors that contribute to respiratory infection and colonization in CMC.

### 4.1. Children with Tracheostomy

Tracheostomy is a life-saving intervention [33] performed in children with multiple chronic conditions requiring complex and ongoing medical care [34,35]. Over the last five decades, the indications for pediatric tracheostomy have shifted significantly. Historically, severe upper airway infections, such as diphtheria, croup, and epiglottitis, were the leading causes of tracheostomy. Today, chronic respiratory failure in young children has become the primary indication [36,37]. A multicenter epidemiological study conducted in Spain (2008–2009) involving 249 pediatric patients identified prolonged ventilation as the most common indication (62.6%), followed by acquired subglottic stenosis (13.6%), congenital or acquired craniofacial anomalies (10%), and congenital airway anomalies (9.6%). Most of these patients had underlying neurological or respiratory chronic diseases [38].

Infection remains the most common complication associated with tracheostomy [39]. Under normal circumstances, the trachea has physiological defense mechanisms, including mucus production, ciliary function, and immunoglobulin secretion, that maintain a balanced microbial environment. However, alterations in the tracheal epithelium disrupt these defenses, leading to changes in mucus production, impaired ciliary movement, and reduced secretion of immunoglobulins [40]. Tracheostomy bypasses natural protective barriers, such as the nose and mouth, creating a direct conduit for external pathogens into the lower airways [41]. These anatomical changes increase the risk of colonization and infection by various microorganisms, a finding supported by the frequent detection of pathogenic microorganisms in the airways of children with tracheostomy [41].

Proper hygiene, regular site care, and routine surveillance are essential to minimize infection risks in these children [42]. Pathogen detection is typically performed through endotracheal aspirates, which have comparable sensitivity to bronchoalveolar lavage for identifying *Pseudomonas aeruginosa* and *Staphylococcus aureus* [43]. A quantitative microbial analysis of ≥10^6^ CFU/mL indicates significant colonization, with a specificity of 90% and sensitivity of 50% [44]. Despite these diagnostic advances, data on bacterial colonization in tracheostomized children remain limited [45,46].

Tracheostomy tubes may be colonized by endogenous bacteria from the patient’s oral microbiota or by exogenous bacteria introduced from the environment [47]. Even with meticulous care, most children with tracheostomies become colonized with potentially pathogenic bacteria [48,49]. Bacterial biofilms, consisting of live bacteria embedded in a protective matrix, are frequently observed on the internal surfaces of tracheostomy tubes. These biofilms pose significant challenges, given they decrease antibiotic efficacy, slow bacterial growth, and block phagocytic activity [50,51].

Studies indicate that *Staphylococcus aureus* and *Pseudomonas aeruginosa* are the most common pathogens in tracheostomized children [43,45,46]. Ventilated patients, particularly those hospitalized for extended periods in intensive care units, show a higher prevalence of multidrug-resistant organisms like MRSA due to frequent antibiotic use [52,53]. *Pseudomonas aeruginosa* colonization is more prevalent in endotracheally ventilated patients, whereas *Staphylococcus aureus* is more common in tracheostomized individuals [47].

In addition to these pathogens, *Klebsiella pneumoniae*, *Escherichia coli*, and *Acinetobacter baumannii* are often acquired during rehabilitation after discharge from intensive care [54]. Resistance patterns are concerning; for example, *Pseudomonas aeruginosa* shows high resistance to carbapenems, while *Staphylococcus aureus* frequently resists methicillin and fluoroquinolones [54,55]. Other less prevalent bacteria include *Stenotrophomonas maltophila*, *Serratia marcescens*, and *Citrobacter freundii*, which show varying degrees of susceptibility to tested antibiotics [56,57,58].

The persistent colonization of the airways by *Pseudomonas aeruginosa* is not consistently associated with increased respiratory infections, making the treatment of asymptomatic colonization a debated topic [59,60]. However, a retrospective cohort study demonstrated that pre-tracheostomy colonization with *Pseudomonas aeruginosa* is associated with chronic colonization in the following two years, suggesting that early eradication might be beneficial [61]. Similarly, targeted nebulized antibiotic therapy after systemic antibiotics has been shown to reduce hospitalizations and intensive care unit stays in children with persistent bacterial colonization and respiratory exacerbations [62]. However, the precise relationship between colonization and exacerbations remains unclear [63,64,65].

When treating tracheostomized children with symptoms of respiratory infection, empirical antibiotic therapy should target *Pseudomonas aeruginosa* and *Staphylococcus aureus*, with MRSA considered based on the duration of tracheostomy and local prevalence rates [45]. Antibiotic selection should also account for the pathogens identified in tracheal cultures within the preceding 7–30 days [66]. However, studies suggest that bacteria isolated during acute exacerbations often differ from those identified in previous aspirates, indicating a dynamic interaction between colonization and infection triggers [41]. This evolving understanding underscores the need for individualized and evidence-based management strategies to address the challenges of airway colonization and infection in tracheostomized children.

### 4.2. Children with Severe Neurological Impairment

Patients with complex neurological disabilities frequently experience respiratory complications, which are significant contributors to morbidity and mortality in this population [67,68]. Among these patients, individuals with cerebral disorders are particularly prone to recurrent aspiration of saliva, solids, and liquids. Dysphagia is a common issue arising from oromotor dysfunctions, anatomical abnormalities, abnormal neurological development, or esophageal motility disorders. This condition is often exacerbated by the high prevalence of gastroesophageal reflux disease in this population [69].

A survey of 1357 children from the Northern Ireland Cerebral Palsy Register (1992–2009) reported that 43% of children with cerebral palsy experienced dysphagia to some extent [70]. While the exact prevalence of pulmonary aspiration in cerebral palsy is unknown, aspiration pneumonia is a frequent cause of hospitalization. Repeated inhalation events impair mucociliary clearance, leading to recurrent lower airway infections, chronic inflammation, and long-term damage such as bronchiectasis, atelectasis, and fibrosis [71]. Consequently, pneumonia is a leading cause of hospital admissions, intensive care utilization, and death in this population [72].

The severity of respiratory complications often depends on the extent of the neurological impairment. Bulbar dysfunction, common in these patients, limits the ability to maintain glottal patency and produce an effective cough. This impairment is compounded by diminished sensory responses and altered irritant receptor sensitivity. Additionally, many children have kyphoscoliosis or other thoracic cage deformities, which contribute to restrictive lung disease and increased respiratory effort [23].

These factors collectively result in significant respiratory impairment, as illustrated by a US study of severely disabled children, which found that pneumonia accounted for 77% of deaths in this population [68].

A prospective pediatric study conducted in the UK (1999–2005) investigated airway bacterial flora in ventilated children with cerebral palsy admitted to intensive care. Among 53 children studied, 47 (89%) had abnormal bacterial flora, most commonly *Pseudomonas aeruginosa* and *Klebsiella* spp., with 47% of isolates being resistant bacteria. The rate of airway colonization by Gram-negative anaerobic bacteria was nearly double in children with cerebral palsy (89%) compared to healthy pediatric patients (55%) [73].

Neurological impairments may influence bacterial colonization by altering airway receptor dynamics. Under normal conditions, a fibronectin layer protects the receptors in the upper airways. In patients with long-term neurological illness, chronic inflammation triggers the production of elastases, which degrade the fibronectin barrier, exposing receptors that favor the adherence of Gram-negative bacteria [74,75]. In pediatric patients with severe neurological impairment, pathogens such as *Pseudomonas aeruginosa*, *Staphylococcus aureus*, and *Enterobacteriaceae* play critical roles in pneumonia development [76]. Children with cerebral palsy colonized by Gram-negative bacteria or *Pseudomonas aeruginosa* are at higher risk of admission to pediatric intensive care units, prolonged hospital stays, and intubations [77]. A large prospective study of children with community-acquired pneumonia showed that those with neurological impairment were older (median 4.2 years) than children with non-neurological underlying conditions (median 2.7 years) or no underlying conditions (median 1.8 years). Children with neurological disorders were significantly less likely to have detectable respiratory pathogens (69.4%) compared to those with non-neurological underlying conditions (84.4%) or no underlying conditions (81.4%). Among viruses, respiratory syncytial virus, adenovirus, and rhinovirus were less frequently detected in children with neurological impairments. While bacterial detection rates were similar across groups, *Streptococcus pneumoniae* was significantly more prevalent in children without underlying conditions (5.1%) compared to those with neurological impairments (1.9%) or non-neurological conditions (2.1%) [78].

Children with severe neurological impairment often experience respiratory complications, which significantly contribute to morbidity and mortality [67,68]. Dysphagia, aspiration of saliva, and gastroesophageal reflux disease are prevalent, leading to recurrent respiratory infections and long-term lung damage, including bronchiectasis and fibrosis [69,70,71]. Pneumonia is among the most common causes of hospitalization and death in this population [72]. A prospective study revealed higher rates of airway colonization with resistant Gram-negative bacteria in children with cerebral palsy compared to healthy peers [65]. Chronic inflammation and impaired mucosal defenses further predispose these patients to colonization by organisms like *Pseudomonas aeruginosa* and *Klebsiella* spp. [66,67,68]. These pathogens are strongly associated with increased admissions to pediatric intensive care units and prolonged hospital stays [69].

These findings underscore the unique respiratory challenges faced by patients with complex neurological disabilities, highlighting the need for tailored management strategies to address aspiration, infections, and long-term respiratory health.

### 4.3. Patients with Neuromuscular Disease

Respiratory insufficiency in patients with neuromuscular diseases is typically progressive and influenced by the underlying condition [79,80]. These diseases often manifest initially as low tidal volume breathing and sleep-related hypoventilation, exacerbated by upper airway hypotonia that leads to obstruction. The cough reflex is frequently weakened or absent, further impairing airway clearance. Effective coughing requires deep inhalation, temporary glottal closure to increase intrathoracic pressure, and a forceful glottic opening coordinated with abdominal contraction to expel air. Peak cough flow (PCF), measured using a peak flow meter, is a critical indicator of cough strength. In children over 12 years of age, a PCF < 270 L/min indicates a weak cough, while values < 160 L/min are associated with an increased risk of severe respiratory infections and hospital admissions [81]. For non-collaborative patients, impaired airway clearance is diagnosed based on caregiver and provider observations of a chronic productive cough and recurrent pneumonia. Children with compromised inspiratory or expiratory muscles commonly exhibit reduced PCF, worsening their ability to clear secretions [80].

Mucociliary clearance is often impaired due to low tidal volumes during breathing, leading to chronic retention of airway secretions. This stagnation can alter the pulmonary microbiome in patients with neuromuscular diseases, even in the absence of primary lung disease, thereby increasing the risk of respiratory infections and chronic inflammation [82]. A retrospective study by Stehling et al. demonstrated that declining cough strength and lung volume predisposed patients with neuromuscular diseases to upper airway colonization [83]. Among 77 children studied, none exhibited symptoms of airway infection or were receiving antibiotics, yet 51% (39/77) harbored potentially pathogenic organisms. The most commonly identified microorganism was *Candida albicans*, followed by Staphylococcus aureus and Gram-negative bacteria. Colonized patients showed significant reductions in both forced vital capacity (FVC) and PCF. Additionally, the use of non-invasive ventilation (NIV) or a mechanical-assisted cough was strongly associated with upper airway colonization. Patients colonized by *Staphylococcus aureus* and Gram-negative bacteria exhibited significantly lower lung volumes and more frequent use of NIV or a mechanical-assisted cough compared to those colonized by other pathogens [83]. In another study, of 128 samples from patients with neuromuscular diseases, 19 (15%) tested positive for *Pseudomonas aeruginosa*, with a higher prevalence in patients with tracheostomies [84]. Although no significant relationship was observed between the type of antibiotic and clinical outcomes, epidemiological data suggest that pathogen-specific antibiotics, particularly targeting *Pseudomonas aeruginosa*, may lead to faster clinical improvement [84]. A small retrospective study of 15 pediatric patients with neuromuscular diseases (*n* = 5 Duchenne muscular dystrophy, *n* = 6 amyotrophic lateral sclerosis, *n* = 2 spinal muscular atrophy type 2, and *n* = 2 congenital myopathy) evaluated nebulized antibiotic therapy against Gram-negative bacteria [85]. Pathogens included *Pseudomonas aeruginosa* (*n* = 9), *Klebsiella pneumoniae* (*n* = 1), *Acinetobacter baumannii* (*n* = 1), and mixed pathogens in four patients. All patients had severe restrictive lung disease and were mechanically ventilated. Nebulized colomycin eradicated pathogens in five patients and reduced colony-forming units in others [85].

Further research is required to confirm the benefits of nebulized antibiotics in reducing colonization by multidrug-resistant bacteria. Successful eradication of these pathogens could lower respiratory infection rates and hospitalizations, improving outcomes for children with neuromuscular diseases [86,87].

## 5. Therapeutic Options for Airway Colonization in Children with Medical Complexity (CMC)

CMC often face significant respiratory challenges, including impaired mucociliary clearance, chronic low tidal volume, and recurrent respiratory infections [1,2]. Effective management typically involves airway clearance therapies (ACT) and the administration of systemic or nebulized antibiotics, which can be tailored to the specific needs of these children. The management of respiratory infections in CMC requires a nuanced approach that balances the need for effective infection control with the risks associated with prolonged antibiotic use [88].

ACT and antibiotic therapies are complementary strategies that target different aspects of respiratory care. While ACT improves the physical clearance of secretions and optimizes lung function, antibiotics address the microbial component of respiratory disease. Key challenges remain in ensuring adherence to ACT, particularly in younger children and those with cognitive impairments. By combining ACT with targeted antibiotic therapy, clinicians can significantly reduce the respiratory burden in CMC, improving both clinical outcomes and patient quality of life. Tailored multidisciplinary care remains essential to address the complex and multifactorial nature of respiratory complications in this vulnerable population.

### 5.1. Airway Clearance Therapies (ACT)

Airway clearance therapies play a crucial role in reducing the respiratory burden in CMC by addressing chronic low tidal volumes and impaired mucociliary clearance. The primary objectives of ACT are to remove secretions from the peripheral and central airways, prevent atelectasis, improve lung compliance, and maintain chest wall mobility [89,90]. These therapies are especially beneficial in children who lack effective natural clearance mechanisms due to neuromuscular or neurological impairments or in conditions where secretions are dense and viscous.

Mechanical insufflation–exsufflation, also known as a cough assistant, is highly effective in clearing the central airways [89,91,92]. It applies positive pressure to inflate the lungs, followed by a rapid switch to negative pressure to simulate a natural cough reflex. It is particularly beneficial for patients with neuromuscular diseases, who often lack the ability to generate an effective cough on their own [89,91,92].

Intrapulmonary percussive ventilation (IPV) is another advanced technique that generates high-velocity positive pressure oscillations, producing vibrations in the peripheral airways [93]. This helps mobilize secretions from the peripheral to central airways. A randomized controlled trial comparing IPV with incentive spirometry in 18 neuromuscular patients showed that IPV significantly reduced episodes of pneumonia or bronchitis, days of antibiotic use, and hospitalizations [93]. These findings highlight the potential of IPV to reduce the burden of infections and improve respiratory outcomes.

The forced exhalation technique encourages children to exhale forcefully and in a controlled manner, facilitating the removal of secretions [94]. It is particularly useful in patients with neurological damage or dense, viscous secretions due to underlying pathologies [94].

For very young children or those with significant cognitive impairments, adherence to ACT can be challenging. Tailoring techniques to meet the specific needs of the child and providing comprehensive caregiver training are essential. Caregivers play a pivotal role in the daily management of these therapies, which require consistent commitment.

### 5.2. Systemic or Nebulized Antibiotics

Respiratory infections in CMC present significant therapeutic challenges due to their recurrent nature, the involvement of multidrug-resistant pathogens, and the risk of systemic side effects. Both systemic and nebulized antibiotics are critical tools in the management of these infections, and the choice of therapy depends on the identified pathogens, the severity of the infection, the patient’s clinical response, and the potential for side effects [95].

Systemic antibiotics are typically used for severe or disseminated infections and include beta-lactams, fluoroquinolones, aminoglycosides, or macrolides. Antibiotic selection is guided by microbiological cultures and local resistance patterns to ensure targeted therapy [96]. This approach is vital to reduce inappropriate antibiotic use and prevent the development of antimicrobial resistance, particularly in patients already colonized by multidrug-resistant organisms. However, systemic antibiotics can be associated with significant side effects, including gastrointestinal disturbances, allergic reactions, and renal toxicity.

Nebulized antibiotics offer a more localized approach to treating respiratory infections by delivering high concentrations of the drug directly to the respiratory tract while minimizing systemic exposure and side effects [97]. This strategy, widely used in cystic fibrosis, has been adapted for use in CMC. Nebulized antibiotics such as colistin and tobramycin are effective in reducing the density of *Pseudomonas aeruginosa*, preventing exacerbations, and improving airway health [98,99,100].

Atag et al. demonstrated that nebulized antibiotics significantly reduced hospital admissions, intensive care unit stays, and bacterial loads in tracheostomized patients with persistent airway colonization [62]. In their study, 22 patients were treated with gentamicin (63.6%) or colistin (36.4%) for an average duration of three months (range: 2–5 months). Importantly, no significant side effects were reported, highlighting the safety and efficacy of this approach [62]. While nebulized antibiotics are highly effective for localized respiratory infections, systemic antibiotics remain essential for severe or disseminated infections. In many cases, a combination of systemic and nebulized antibiotics is used to maximize therapeutic efficacy while minimizing the risk of resistance and adverse effects. This dual approach allows clinicians to target infections comprehensively while preserving patient safety.

## 6. Clinical Management of Children with Medical Complexity (CMC)

CMC present a unique challenge for pediatricians and healthcare systems due to their demanding healthcare needs, which place a significant burden on caregivers and society. Although CMC represent a small fraction of the pediatric population, they account for a disproportionately high share of healthcare utilization, including hospitalizations, intensive care admissions, and healthcare expenditures [4]. Managing these medically fragile patients requires coordination across multiple specialists and settings, including hospitals and home care, as well as the application of specialized skills [101]. Respiratory complications are among the most significant issues faced by CMC, driven by impaired respiration, ineffective cough, and pulmonary aspiration. These factors frequently lead to airway colonization by multidrug-resistant bacteria, a risk that is further heightened by the use of life-saving devices such as tracheostomies.

Although microbiological examinations are critical for targeting infections, the relationship between airway colonization and respiratory exacerbations remains unclear. This gap in understanding complicates clinical decision-making, as there are currently no specific evidence-based guidelines for managing respiratory colonization and infections in CMC. Clinicians must often rely on a combination of patient history, local microbial epidemiology, and clinical presentation to make decisions, such as initiating eradication therapies, which in some cases may improve respiratory conditions. Table 1 summarizes a proactive and structured approach that aims to minimize respiratory complications and improve outcomes in CMC.

Several unanswered questions remain in the management of airway colonization and infections in CMC. Future research should focus on clarifying the relationship between airway colonization and the risk of exacerbations. Understanding which pathogens or colonization markers are most predictive of clinical deterioration could improve risk stratification and inform targeted treatment strategies. There is also an urgent need to develop specific guidelines for managing respiratory colonization and infections in CMC. These should address when to treat colonization, when eradication is appropriate, and how best to use systemic and nebulized antibiotics to balance efficacy and safety. Investigating the potential of eradication therapies is another critical area for exploration. Studies should evaluate whether removing specific pathogens from the airways of CMC patients can reduce respiratory infections, prevent hospitalizations, and improve long-term outcomes. Advances in diagnostic technologies, such as molecular diagnostics and rapid point-of-care testing, could enable earlier and more accurate identification of pathogens, allowing for more timely and precise interventions. Research should also focus on improving airway clearance strategies, exploring how these can be tailored to individual needs and combined with other interventions like nebulized antibiotics. Innovations in child-friendly devices and protocols may enhance adherence, which is often challenging in younger or cognitively impaired patients. The growing prevalence of multidrug-resistant organisms highlights the need for new antimicrobial agents and preventive strategies, such as biofilm inhibitors, antimicrobial-coated devices, and the potential role of probiotics. Support for caregivers is another vital aspect. Studies should assess how education, training, and access to resources can empower caregivers to manage the complex respiratory needs of CMC, potentially improving outcomes and reducing stress. Early interventions are also likely to play a key role in preventing respiratory decline. Longitudinal research tracking CMC from early childhood to adolescence could provide critical insights into the natural history of respiratory complications and the effectiveness of preemptive strategies. Moreover, advancements in nanomedicine and precision medicine are paving the way for innovative approaches to managing airway colonization in CMC [102,103,104]. Nanotechnology offers the potential for targeted drug delivery systems that can directly address pathogens within biofilms or specific respiratory sites, improving efficacy while minimizing systemic exposure and side effects [102]. Precision medicine, with its focus on individualized care, leverages genomic and microbiome profiling to tailor antibiotic and therapeutic interventions based on the specific pathogens and resistance patterns identified in each patient [103]. These approaches aim to reduce reliance on broad-spectrum antibiotics, thereby curbing antimicrobial resistance. Furthermore, the development of antimicrobial-coated medical devices and biofilm inhibitors could significantly lower the risk of colonization in children reliant on tracheostomies or ventilatory support [104,105]. These emerging strategies represent promising avenues for enhancing the management and long-term outcomes of airway colonization in CMC, aligning with ongoing efforts to optimize patient-centered care.

In addition to clinical outcomes, improving the quality of life for CMC and their families should remain a central goal. Caregivers often face emotional, physical, and financial burdens due to the constant demands of managing their child’s complex needs [106]. This stress can lead to burnout, depression, and decreased capacity to adhere to rigorous treatment regimens, ultimately impacting the child’s health outcomes. For example, inconsistent administration of ACT or medications due to caregiver fatigue can exacerbate respiratory complications. Incorporating robust caregiver support mechanisms is essential to mitigate these challenges. Providing access to respite care, training programs, peer support groups, and mental health resources can improve caregiver well-being and their ability to manage the child’s needs effectively [107]. Additionally, involving caregivers in the development of tailored care plans and decision-making processes fosters collaboration, enhances adherence, and ensures that management strategies align with family capabilities and preferences [107]. A more holistic approach to managing CMC, which addresses both the child’s medical needs and caregiver support, is crucial for improving outcomes and enhancing the overall quality of life for families navigating these challenges. Future research should address the psychological, social, and economic impacts of respiratory complications and their management. By advancing our understanding and addressing these priorities, healthcare systems can develop innovative diagnostic and therapeutic strategies that enhance patient outcomes, reduce the burden on caregivers, and improve the overall quality of life for this vulnerable population.

## 7. Conclusions

The management of CMC requires a multidisciplinary and individualized approach to address their diverse and interconnected healthcare needs. Respiratory complications, particularly airway colonization and recurrent infections, represent a significant challenge, underscoring the importance of proactive screening, tailored interventions, and family-centered care. Future research should prioritize understanding the relationship between airway colonization and clinical outcomes, developing evidence-based guidelines, and improving therapeutic strategies to enhance the quality of life and long-term outcomes for this vulnerable population.

## Figures and Tables

**Table 1:** Approach aims to minimize respiratory complications and improve outcomes in children with medical complexity (CMC).

Algorithm for Managing Children with CMC with Respiratory Problems
**1.** **Initial Evaluation** **Clinical Assessment:** ◦ Evaluate symptoms: cough, wheezing, dyspnea, nocturnal symptoms. ◦ Assess history of previous respiratory infections, hospitalizations, or antibiotic use. ◦ Identify risk factors: tracheostomy, mechanical ventilation, neuromuscular disorders, or gastroesophageal reflux. **Baseline Investigations:** ◦ Pulse oximetry: assess oxygen saturation. ◦ Capnography: measure end-tidal CO_2_ for hypoventilation. ◦ Chest X-ray or computed tomography scan if structural abnormalities are suspected. ◦ Complete blood count (CBC) and inflammatory markers for infection. **2.** **Screening for Respiratory Complications** **Routine Airway Pathogen Screening:** ◦ Conduct periodic sputum cultures, tracheal aspirates, or nasopharyngeal swabs for airway colonization. ◦ Identify pathogens like *Pseudomonas aeruginosa*, *Staphylococcus aureus*, or multidrug-resistant organisms. **Nocturnal Hypoventilation Screening:** ◦ Perform overnight pulse oximetry or capnography. ◦ Polysomnography for suspected sleep-disordered breathing or hypoventilation. ◦ Assess for central or obstructive sleep apnea. **3.** **Management of Active Respiratory Problems** **Airway Clearance:** ◦ Initiate airway clearance therapy (ACT) with techniques such as high-frequency chest wall oscillation, positive expiratory pressure (PEP), or mechanical insufflation–exsufflation devices. **Oxygen Therapy:** ◦ Administer supplemental oxygen for hypoxemia, ensuring careful monitoring in children with chronic CO₂ retention. **Antibiotic Therapy:** ◦ Use systemic antibiotics for acute exacerbations guided by culture sensitivity. ◦ Nebulized antibiotics (e.g., tobramycin, colistin) for chronic colonization or resistant pathogens. **Non-Invasive Ventilation (NIV):** ◦ Consider NIV (e.g., BiPAP) in children with hypoventilation or restrictive lung disease. **Advanced Support:** ◦ Tracheostomy care and ventilation adjustments for children with severe respiratory compromise.
**Preventative Steps Before Respiratory Problems Occur**
**1.** **Regular Monitoring** Conduct routine respiratory assessments, including lung function tests if feasible. Monitor growth and nutritional status, as poor nutrition exacerbates respiratory issues. **2.** **Screening Programs** **Airway Colonization:** ◦ Perform regular microbiological assessments to detect and manage early colonization. **Hypoventilation:** ◦ Initiate early polysomnography in children with risk factors like neuromuscular diseases or thoracic deformities. **3.** **Proactive Interventions** **Immunizations:** ◦ Ensure up-to-date vaccinations, including influenza and pneumococcal vaccines. **Airway Clearance:** ◦ Educate caregivers on daily ACT to prevent secretion build-up. **Gastroesophageal Reflux Disease Management:** ◦ Manage reflux with lifestyle changes, medications, or surgical interventions to reduce aspiration risk. **Aspiration Precautions:** ◦ Perform swallowing assessments and use thickened feeds if indicated. **4.** **Multidisciplinary Approach** Collaborate with pulmonologists, respiratory therapists, nutritionists, and physiotherapists. Develop individualized care plans tailored to the child’s specific needs. **5.** **Caregiver Training** Train caregivers in recognizing early signs of respiratory distress, proper ACT techniques, and emergency responses. **6.** **Preventative Therapies** Nebulized saline or bronchodilators as maintenance therapy for high-risk children. Prophylactic antibiotics in select cases with recurrent infections.
**Key Considerations**
• Adjust care plans based on evolving clinical status and new findings. • Incorporate family input and ensure alignment with the child’s overall care goals.

## Supplementary Materials

The following supporting information can be downloaded at: https://www.mdpi.com/article/10.3390/jcm14030848/s1, Supplementary Materials S1: Definitional criteria of children with medical complexity (CMC) divided by domain (S1). Reference [3] is cited in the Supplementary Materials.

## References

[1] Agostiniani R., Nanni L., Langiano T. (2014). Children with medical complexity: The change in the pediatric epidemiology. J. Pediatr. Neonat. Individ. Med..

[2] Gallo M., Agostiniani R., Pintus R., Fanos V. (2021). The child with medical complexity. Ital. J. Pediatr..

[3] Millar K., Rodd C., Rempel G., Cohen E., Sibley K.M., Garland A. (2024). The Clinical Definition of Children with Medical Complexity: A Modified Delphi Study. Pediatrics.

[4] Leyenaar J.K., Schaefer A.P., Freyleue S.D., Austin A.M., Simon T.D., Van Cleave J., Moen E.L., O’malley A.J., Goodman D.C. (2022). Prevalence of Children with Medical Complexity and Associations with Health Care Utilization and In-Hospital Mortality. JAMA Pediatr..

[5] Teicher J., Moore C., Esser K., Weiser N., Arje D., Cohen E., Orkin J. (2023). The Experience of Parental Caregiving for Children With Medical Complexity. Clin. Pediatr..

[6] Pordes E., Gordon J., Sanders L.M., Cohen E. (2018). Models of Care Delivery for Children With Medical Complexity. Pediatrics.

[7] Cohen E., Berry J.G., Camacho X., Anderson G., Wodchis W., Guttmann A. (2012). Patterns and costs of health care use of children with medical complexity. Pediatrics.

[8] Berry J.G., Hall M., Cohen E., O’Neill M., Feudtner C. (2015). Ways to Identify Children with Medical Complexity and the Importance of Why. J. Pediatr..

[9] Moon M.R. (2023). Everyday Ethics in the Clinical Practice of Pediatrics and Young Adult Medicine, an Issue of Pediatric Clinics of North America.

[10] Oliveira P.V., Enes C.C., Nucci L.B. (2023). How are children with medical complexity being identified in epidemiological studies? A systematic review. World J. Pediatr..

[11] Suzuki Y. (2008). New scoring system for patients with severe motor and intellectual disabilities, medical care dependent group. Jpn. J. Sev. Mot. Intellect. Disabil..

[12] Simon T.D., Cawthon M.L., Stanford S., Popalisky J., Lyons D., Woodcox P., Hood M., Chen A.Y., Mangione-Smith R. (2014). Pediatric medical complexity algorithm: A new method to stratify children by medical complexity. Pediatrics.

[13] Cohen E., Kuo D.Z., Agrawal R., Berry J.G., Bhagat S.K., Simon T.D., Srivastava R. (2011). Children with medical complexity: An emerging population for clinical and research initiatives. Pediatrics.

[14] Feudtner C., Christakis D.A., Connell F.A. (2000). Pediatric deaths attributable to complex chronic conditions: A population-based study of Washington State, 1980–1997. Pediatrics.

[15] Feudtner C., Feinstein J.A., Zhong W., Hall M., Dai D. (2014). Pediatric complex chronic conditions classification system version 2: Updated for ICD-10 and complex medical technology dependence and transplantation. BMC Pediatr..

[16] Hughes J.S., Averill R.F., Eisenhandler J., Goldfield N.I., Muldoon J., Neff J.M., Gay J.C. (2004). Clinical Risk Groups (CRGs): A classification system for risk-adjusted capitation-based payment and health care management. Med. Care.

[17] Berry J.G., Agrawal R., Kuo D.Z., Cohen E., Risko W., Hall M., Casey P., Gordon J., Srivastava R. (2011). Characteristics of hospitalizations for patients who use a structured clinical care program for children with medical complexity. J. Pediatr..

[18] Kuo D.Z., Cohen E., Agrawal R., Berry J.G., Casey P.H. (2011). A national profile of caregiver challenges among more medically complex children with special health care needs. Arch. Pediatr. Adolesc. Med..

[19] Edwards J.D., Houtrow A.J., Vasilevskis E.E., Rehm R.S., Markovitz B.P., Graham R.J., Dudley R.A. (2012). Chronic conditions among children admitted to U.S. pediatric intensive care units: Their prevalence and impact on risk for mortality and prolonged length of stay. Crit. Care Med..

[20] Feudtner C., Hays R.M., Haynes G., Geyer J.R., Neff J.M., Koepsell T.D. (2001). Deaths attributed to pediatric complex chronic conditions: National trends and implications for supportive care services. Pediatrics.

[21] Berry J.G., Poduri A., Bonkowsky J.L., Zhou J., Graham D.A., Welch C., Putney H., Srivastava R. (2012). Trends in resource utilization by children with neurological impairment in the United States inpatient health care system: A repeat cross-sectional study. PLoS Med..

[22] Tzeng A.C., Bach J.R. (2000). Prevention of pulmonary morbidity for patients with neuromuscular disease. Chest..

[23] Esposito S., Ricci G., Gobbi R., Vicini C., Caramelli F., Pizzi S., Fadda A., Ferro S., Plazzi G. (2022). Diagnostic and Therapeutic Approach to Children and Adolescents with Obstructive Sleep Apnea Syndrome (OSA): Recommendations in Emilia-Romagna Region, Italy. Life.

[24] Mussi N., Forestiero R., Zambelli G., Rossi L., Caramia M.R., Fainardi V., Esposito S. (2023). The First-Line Approach in Children with Obstructive Sleep Apnea Syndrome (OSA). J. Clin. Med..

[25] Patria M.F., Longhi B., Lelii M., Tagliabue C., Lavelli M., Galeone C., Principi N., Esposito S. (2016). Children with recurrent pneumonia and non-cystic fibrosis bronchiectasis. Ital. J. Pediatr..

[26] Patria M.F., Ghislanzoni S., Macchini F., Lelii M., Mori A., Leva E., Principi N., Esposito S. (2017). Respiratory Morbidity in Children with Repaired Congenital Esophageal Atresia with or without Tracheoesophageal Fistula. Int. J. Environ. Res. Public Health.

[27] Orishchak O., Moise A., Al-Osamey F., Kaspy K., Daniel S.J. (2024). Lipid-laden macrophage index as a marker of aspiration in children, is it reliable? A scoping review. Int. J. Pediatr. Otorhinolaryngol..

[28] Goodarzy B., Rahmani E., Farrokhi M., Tavakoli R., Moghadam Fard A., Ranjbaran Ghaleh M., Ghalichebaf Yazdi Y., Amani Beni R., Ghadirzadeh E., Afrazeh F. (2024). Diagnostic Value of Chest Computed Tomography Scan for Identification of Foreign Body Aspiration in Children: A Systematic Review and Meta-analysis. Arch. Acad. Emerg. Med..

[29] DellaBadia K., Tauber D. (2021). Respiratory concerns in children with medical complexity. Curr. Probl. Pediatr. Adolesc. Health Care.

[30] Salera S., Menni F., Moggio M., Guez S., Sciacco M., Esposito S. (2017). Nutritional Challenges in Duchenne Muscular Dystrophy. Nutrients.

[31] Sethi S., Murphy T.F. (2008). Infection in the pathogenesis and course of chronic obstructive pulmonary disease. N. Engl. J. Med..

[32] Mourani P.M., Sontag M.K., Williamson K.M., Harris J.K., Reeder R., Locandro C., Carpenter T.C., Maddux A.B., Ziegler K., Simões E.A. (2021). Temporal airway microbiome changes related to ventilator-associated pneumonia in children. Eur. Respir. J..

[33] Al-Samri M., Mitchell I., Drummond D.S., Bjornson C. (2010). Tracheostomy in children: A population-based experience over 17 years. Pediatr. Pulmonol..

[34] Graf J.M., Montagnino B.A., Hueckel R., McPherson M.L. (2008). Pediatric tracheostomies: A recent experience from one academic center. Pediatr. Crit. Care Med..

[35] Trachsel D., Hammer J. (2006). Indications for tracheostomy in children. Paediatr. Respir. Rev..

[36] Liu C., Heffernan C., Saluja S., Yuan J., Paine M., Oyemwense N., Berry J., Roberson D. (2014). Indications, Hospital Course, and Complexity of Patients Undergoing Tracheostomy at a Tertiary Care Pediatric Hospital. Otolaryngol. Head Neck Surg..

[37] Berry J.G., Graham D.A., Graham R.J., Zhou J., Putney H.L., O’Brien J.E., Roberson D.W., Goldmann D.A. (2009). Predictors of clinical outcomes and hospital resource use of children after tracheotomy. Pediatrics.

[38] Pérez-Ruiz E., Caro P., Pérez-Frías J., Cols M., Barrio I., Torrent A., García M., Asensio O., Pastor M.D., Luna C. (2012). Paediatric patients with a tracheostomy: A multicentre epidemiological study. Eur. Respir. J..

[39] Carr M.M., Poje C.P., Kingston L., Kielma D., Heard C. (2001). Complications in pediatric tracheostomies. Laryngoscope.

[40] Rao A.R., Splaingard M.S., Gershan W.M., Havens P.L., Thill A., Barbieri J.T. (2005). Detection of *Pseudomonas aeruginosa* type III antibodies in children with tracheostomies. Pediatr. Pulmonol..

[41] Cline J.M., Woods C.R., Ervin S.E., Rubin B.K., Kirse D.J. (2012). Surveillance tracheal aspirate cultures do not reliably predict bacteria cultured at the time of an acute respiratory infection in children with tracheostomy tubes. Chest.

[42] Jaryszak E.M., Shah R.K., Amling J., Peña M.T. (2011). Pediatric tracheotomy wound complications: Incidence and significance. Arch. Otolaryngol. Head Neck Surg..

[43] McLaren D., Chitakis M., Burns H., Kapur N. (2021). Airway Microbiology in Tracheostomized Children. Respir. Care.

[44] Wu C.L., Yang D.I., Wang N.Y., Kuo H.T., Chen P.Z. (2002). Quantitative culture of endotracheal aspirates in the diagnosis of ventilator-associated pneumonia in patients with treatment failure. Chest.

[45] McCaleb R., Warren R.H., Willis D., Maples H.D., Bai S., O’Brien C.E. (2016). Description of Respiratory Microbiology of Children With Long-Term Tracheostomies. Respir. Care.

[46] Tan C.-Y., Chiu N.-C., Lee K.-S., Chi H., Huang F.-Y., Huang D.T.-N., Chang L., Kung Y.-H., Huang C.-Y. (2020). Respiratory tract infections in children with tracheostomy. J. Microbiol. Immunol. Infect..

[47] Morar P., Singh V., Makura Z., Jones A., Baines P., Selby A., Sarginson R., Hughes J., van Saene R. (2002). Differing pathways of lower airway colonization and infection according to mode of ventilation (endotracheal vs. tracheotomy). Arch. Otolaryngol. Head Neck Surg..

[48] Brook I. (1979). Bacterial colonization, tracheobronchitis, and pneumonia following tracheostomy and long-term intubation in pediatric patients. Chest.

[49] Niederman M.S., Ferranti R.D., Zeigler A., Merrill W.W., Reynolds H.Y. (1984). Respiratory infection complicating long-term tracheostomy. The implication of persistent gram-negative tracheobronchial colonization. Chest.

[50] Perkins J., Mouzakes J., Pereira R., Manning S. (2004). Bacterial biofilm presence in pediatric tracheotomy tubes. Arch. Otolaryngol. Head Neck Surg..

[51] Solomon D.H., Wobb J., Buttaro B.A., Truant A., Soliman A.M.S. (2009). Characterization of bacterial biofilms on tracheostomy tubes. Laryngoscope.

[52] Havens T.N., Rosen D.A., Rivera-Spoljaric K. (2023). Airway multidrug-resistant organisms in a population of tracheostomy and chronic ventilator-dependent children at a tertiary care pediatric hospital. Pediatr. Pulmonol..

[53] Vickery K., Deva A., Jacombs A., Allan J., Valente P., Gosbell I.B. (2012). Presence of biofilm containing viable multiresistant organisms despite terminal cleaning on clinical surfaces in an intensive care unit. J. Hosp. Infect..

[54] Pozzi M., Pellegrino P., Galbiati S., Granziera M., Locatelli F., Carnovale C., Perrone V., Antoniazzi S., Perrotta C., Strazzer S. (2015). Prevalence of respiratory colonisations and related antibiotic resistances among paediatric tracheostomised patients of a long-term rehabilitation centre in Italy. Eur. J. Clin. Microbiol. Infect. Dis..

[55] Vasconcellos Severo G., Schweiger C., Manica D., Marostica P.J.C. (2023). Tracheostomized children tracheal colonization and antibiotic resistance profile—A STROBE analysis. Eur. Ann. Otorhinolaryngol. Head Neck Dis..

[56] Sanders C.D., Guimbellot J.S., Muhlebach M.S., Lin F.-C., Gilligan P., Esther C.R. (2018). Tracheostomy in children: Epidemiology and clinical outcomes. Pediatr. Pulmonol..

[57] Adler A., Ben-David D., Schwaber M.J., Masarwa S., Schwartz D., Porat N., Kotlovsky T., Shklyar M., Polivkin N., Weinberg I. (2012). Prevalence of Streptococcus pneumoniae in respiratory samples from patients with tracheostomy in a long-term-care facility. J. Clin. Microbiol..

[58] Erdem G., Singh A.K., Brusnahan A.J., Moore A.N., Barson W.J., Leber A., Vidal J.E., Atici S., King S.J. (2018). Pneumococcal colonization among tracheostomy tube dependent children. PLoS ONE.

[59] Lusuardi M., Capelli A., Di Stefano A., Zaccaria S., Balbi B., Donner C.F. (2003). Lower respiratory tract infections in chronic obstructive pulmonary disease outpatients with tracheostomy and persistent colonization by *P. aeruginosa*. Respir. Med..

[60] Kusahara D.M., Friedlander L.T., Peterlini M.A.S., Pedreira M.L.G. (2012). Oral care and oropharyngeal and tracheal colonization by Gram-negative pathogens in children. Nurs. Crit. Care.

[61] Russell C.J., Simon T.D., Neely M.N. (2019). Development of Chronic *Pseudomonas aeruginosa*-Positive Respiratory Cultures in Children with Tracheostomy. Lung.

[62] Atag E., Unal F., Arslan H., Teber B.G., Telhan L., Ersu R., Karakoc F., Oktem S. (2021). The effect of nebulized antibiotics in children with tracheostomy. Int. J. Pediatr. Otorhinolaryngol..

[63] Starner T.D., Zhang N., Kim G., Apicella M.A., McCray P.B. (2006). *Haemophilus influenzae* forms biofilms on airway epithelia: Implications in cystic fibrosis. Am. J. Respir. Crit. Care Med..

[64] Smith E.E., Buckley D.G., Wu Z., Saenphimmachak C., Hoffman L.R., D’argenio D.A., Ramsey B.W., Speert D.P., Moskowitz S.M., Burns J.L. (2006). Genetic adaptation by *Pseudomonas aeruginosa* to the airways of cystic fibrosis patients. Proc. Natl. Acad. Sci. USA.

[65] Sethi S., Evans N., Grant B.J.B., Murphy T.F. (2002). New strains of bacteria and exacerbations of chronic obstructive pulmonary disease. N. Engl. J. Med..

[66] García-Boyano M., Climent Alcalá F.J., Rodríguez Alonso A., García Fernández de Villalta M., Zubiaur Alonso O., Rabanal Retolaza I., Melero I.Q., Calvo C., García L.E. (2024). Pneumonia in Children With Complex Chronic Conditions with Tracheostomy: An Emerging Challenge. Pediatr. Infect. Dis. J..

[67] Seddon P.C., Khan Y. (2003). Respiratory problems in children with neurological impairment. Arch. Dis. Child..

[68] Plioplys A.V. (2003). Survival rates of children with severe neurologic disabilities: A review. Semin. Pediatr. Neurol..

[69] Rogers B. (2004). Feeding method and health outcomes of children with cerebral palsy. J. Pediatr..

[70] Parkes J., Hill N., Platt M.J., Donnelly C. (2010). Oromotor dysfunction and communication impairments in children with cerebral palsy: A register study. Dev. Med. Child. Neurol..

[71] Buu M.C. (2017). Respiratory complications, management and treatments for neuromuscular disease in children. Curr. Opin. Pediatr..

[72] Young N.L., McCormick A.M., Gilbert T., Ayling-Campos A., Burke T., Fehlings D., Wedge J. (2011). Reasons for hospital admissions among youth and young adults with cerebral palsy. Arch. Phys. Med. Rehabil..

[73] Thorburn K., Jardine M., Taylor N., Reilly N., Sarginson R.E., van Saene H.K.F. (2009). Antibiotic-resistant bacteria and infection in children with cerebral palsy requiring mechanical ventilation. Pediatr. Crit. Care Med..

[74] Dal Nogare A.R., Toews G.B., Pierce A.K. (1987). Increased salivary elastase precedes gram-negative bacillary colonization in postoperative patients. Am. Rev. Respir. Dis..

[75] Mobbs K.J., van Saene H.K., Sunderland D., Davies P.D. (1999). Oropharyngeal gram-negative bacillary carriage in chronic obstructive pulmonary disease: Relation to severity of disease. Respir. Med..

[76] Mauritz M.D., von Both U., Dohna-Schwake C., Gille C., Hasan C., Huebner J., Hufnagel M., Knuf M., Liese J.G., Renk H. (2024). Clinical recommendations for the inpatient management of lower respiratory tract infections in children and adolescents with severe neurological impairment in Germany. Eur. J. Pediatr..

[77] Gerdung C.A., Tsang A., Yasseen AS 3rd Armstrong K., McMillan H.J., Kovesi T. (2016). Association Between Chronic Aspiration and Chronic Airway Infection with *Pseudomonas aeruginosa* and Other Gram-Negative Bacteria in Children with Cerebral Palsy. Lung.

[78] Millman A.J., Finelli L., Bramley A.M., Peacock G., Williams D.J., Arnold S.R., Grijalva C.G., Anderson E.J., McCullers J.A., Ampofo K. (2016). Community-Acquired Pneumonia Hospitalization among Children with Neurologic Disorders. J. Pediatr..

[79] Cutrona C., Pede E., De Sanctis R., Coratti G., Tiberi E., Luciano R., Pera M.C., Velli C., Capasso A., Vento G. (2022). Assessing floppy infants: A new module. Eur. J. Pediatr..

[80] Chatwin M., Toussaint M., Gonçalves M.R., Sheers N., Mellies U., Gonzales-Bermejo J., Sancho J., Fauroux B., Andersen T., Hov B. (2018). Airway clearance techniques in neuromuscular disorders: A state of the art review. Respir. Med..

[81] Dohna-Schwake C., Ragette R., Teschler H., Voit T., Mellies U. (2006). Predictors of severe chest infections in pediatric neuromuscular disorders. Neuromuscul. Disord..

[82] Umbrello G., Esposito S. (2016). Microbiota and neurologic diseases: Potential effects of probiotics. J. Transl. Med..

[83] Stehling F., Pieper N., Bouikidis A., Steinmann J., Rath P.-M., Mellies U. (2016). Upper airway microbial colonization in patients with neuromuscular disorders. Respirology.

[84] Gregson E., Thomas L., Elphick H.E. (2021). *Pseudomonas aeruginosa* infection in respiratory samples in children with neurodisability-to treat or not to treat?. Eur. J. Pediatr..

[85] Crescimanno G., Marrone O. (2017). The microbiome and secondary lung disease in neuromuscular patients: Is it time to change our clinical practice?. Respirology.

[86] Plioplys A.V., Kasnicka I. (2011). Nebulized tobramycin: Prevention of pneumonias in patients with severe cerebral palsy. J. Pediatr. Rehabil. Med..

[87] Eckerland M., Bock C., Olivier M., Pichlmaier L., Steindor M., Stehling F. (2019). Reducing the frequency of respiratory tract infections in severe neurological disorders by inhaled antibiotics: A retrospective data analysis. ERJ Open Res..

[88] Fainardi V., Neglia C., Muscarà M., Spaggiari C., Tornesello M., Grandinetti R., Argentiero A., Calderaro A., Esposito S., Pisi G. (2022). Multidrug-Resistant Bacteria in Children and Adolescents with Cystic Fibrosis. Children.

[89] Fainardi V., Longo F., Faverzani S., Tripodi M.C., Chetta A., Pisi G. (2011). Short-term effects of high-frequency chest compression and positive expiratory pressure in patients with cystic fibrosis. J. Clin. Med. Res..

[90] Strickland S.L., Rubin B.K., Drescher G.S., Haas C.F., A O’Malley C., A Volsko T., Branson R.D., Hess D.R. (2013). AARC clinical practice guideline: Effectiveness of nonpharmacologic airway clearance therapies in hospitalized patients. Respir. Care..

[91] Fisher D.F. (2022). Positive Expiratory Pressure Physiotherapy for Airway Clearance in People with Cystic Fibrosis: A Cochrane Review Summary with Commentary. Respir. Care.

[92] Simonds A.K. (2012). Non-Invasive Respiratory Support.

[93] Volsko T.A., Chatburn R.L., El-Khatib M.F. (2020). Equipment for Respiratory Care.

[94] McAlinden B.M., Hough J.L., Kuys S. (2022). Measuring the effects of airway clearance in mechanically ventilated infants and children: A systematic review. Physiotherapy.

[95] Poulakou G., Siakallis G., Tsiodras S., Arfaras-Melainis A., Dimopoulos G. (2017). Nebulized antibiotics in mechanically ventilated patients: Roadmap and challenges. Expert Rev. Anti Infect. Ther..

[96] Fanelli U., Pappalardo M., Chinè V., Gismondi P., Neglia C., Argentiero A., Calderaro A., Prati A., Esposito S. (2020). Role of Artificial Intelligence in Fighting Antimicrobial Resistance in Pediatrics. Antibiotics.

[97] Esposito S., Rosazza C., Sciarrabba C.S., Principi N. (2017). Inhaled Antibiotic Therapy for the Treatment of Upper Respiratory Tract Infections. J. Aerosol Med. Pulm. Drug Deliv..

[98] Gorham J., Taccone F.S., Hites M. (2023). How to Use Nebulized Antibiotics in Severe Respiratory Infections. Antibiotics.

[99] Langton Hewer S.C., Smith S., Rowbotham N.J., Yule A., Smyth A.R. (2023). Antibiotic strategies for eradicating *Pseudomonas aeruginosa* in people with cystic fibrosis. Cochrane Database Syst. Rev..

[100] Ramsey B.W., Pepe M.S., Quan J.M., Otto K.L., Montgomery A.B., Williams-Warren J., Vasiljev-K M., Borowitz D., Bowman C.M., Marshall B.C. (1999). Intermittent administration of inhaled tobramycin in patients with cystic fibrosis. Cystic Fibrosis Inhaled Tobramycin Study Group. N. Engl. J. Med..

[101] Kuo D.Z., Houtrow A.J., Council on Children with Disabilities (2016). Recognition and Management of Medical Complexity. Pediatrics.

[102] Feng X., Shi Y., Zhang Y., Lei F., Ren R., Tang X. (2024). Opportunities and Challenges for Inhalable Nanomedicine Formulations in Respiratory Diseases: A Review. Int. J. Nanomed..

[103] Mac Aogáin M., Tiew P.Y., Jaggi T.K., Narayana J.K., Singh S., Hansbro P.M., Segal L.N., Chotirmall S.H. (2024). Targeting respiratory microbiomes in COPD and bronchiectasis. Expert Rev. Respir. Med..

[104] Bateman R.M., Sharpe M.D., Jagger J.E., Ellis C.G., Solé-Violán J., López-Rodríguez M., Herrera-Ramos E., Ruíz-Hernández J., Borderías L., Horcajada J. (2016). 36th International Symposium on Intensive Care and Emergency Medicine: Brussels, Belgium. 15–18 March 2016. Crit. Care.

[105] Chen X., Ling X., Liu G., Xiao J. (2022). Antimicrobial Coating: Tracheal Tube Application. Int. J. Nanomed..

[106] Yu J.A., Henderson C., Cook S., Ray K. (2020). Family Caregivers of Children With Medical Complexity: Health-Related Quality of Life and Experiences of Care Coordination. Acad. Pediatr..

[107] Suzuki S., Kamibeppu K. (2022). Impact of respite care on health-related quality of life in children with medical complexity: A parent proxy evaluation. J. Pediatr. Nurs..

