# Supplementary material for: Airway Colonization in Children with Medical Complexity: Challenges and Management Strategies
Source: J Clin Med. 2025 Jan 27;14(3):848. doi: 10.3390/jcm14030848 (PMC11818586; doi:10.3390/jcm14030848)
Supplement: Supplementary file 1 [file jcm-14-00848-s001.zip › jcm-3382222-supplementary.pdf]

Supplementary Materials S1. Definitional criteria of CMC divided by domain.

**Diagnostic Conditions**	
	The child must possess a minimum of 1 diagnosed, or unknown but suspected, chronic medical condition.
**Functional limitations**	
2	Over the past 12 mo, the child required a minimum average of 3 h/wk of prescribed, specialized therapy. Note: This therapy may be delivered by, but not limited to, occupational therapy, physiotherapy, psychology, speech language pathology or by caregivers with consultation from therapists.
3	Currently, the child has impactful limitations in VBSFs or in the performance of ADLs.
	Note: Examples of VBSFs include the maintenance of breathing, airway patency, blood circulation, digestion, waste excretion, etc.
	Note: ADLs include eating, bathing, dressing, toileting, transferring, and maintaining continence.
	Note: Depending on the severity of these limitations the child may require a piece of technology that supports or compensates for a limitation in a physiologic function that is: Essential to survival (eg, devices supporting respiration, feeding, or elimination.) OR Necessary for other "life activities and participation" appropriate for chronologic age. (eg, mobility device, transfer device, specialized seating, sensory augmentation devices, communication augmentation devices, etc)
**Health care use**	
4	Currently, the child requires a school nurse to manage care needs for a minimum average of 5 h/wk, assuming the child regularly attends school.
5	Currently, the child requires homecare personnel. Note: Examples of homecare services include a homecare nurse, respite personnel, or health care aide.
6	At any one time over the past 12 mo, the child needed a minimum of 4 interventions for chronic medical issues. Note: These interventions can be pharmacologic (eg, prescription medications) and/or nonpharmacologic (eg, medical technology).
7	Over the past 12 mo, the child needed a minimum of 1 medical intervention with COMPLEX administration at any one time. Note: These interventions can be pharmacologic and/or nonpharmacologic AND must have increased complexity of administration. For example, a complex intervention may include home total parenteral nutrition administered in a central venous line.
8	Over the past 12 mo, the child had a minimum of 3 health care providers involved in their care. Note: Multiple providers with the same specialty from the same practice count as 1 provider.
9	Over the past 12 mo, the child had a minimum of 6 medically necessary outpatient clinic visits beyond what is expected for the child's age.
10	Over the past 12 mo, the child had a minimum of 3 presentations to the emergency department or urgent care center for emergent or urgent medical issues.
11	Over the past 12 mo, the child had a minimum of 2 unplanned, inpatient hospitalizations, independent of the length of the hospitalization.
12	Over the past 12 mo, the child had a minimum of 7 cumulative days in hospital, independent of the number of hospitalizations.
13	Over the past 12 mo, the child had a minimum of 2 readmissions to hospital. Note: Readmissions are defined as an unplanned hospitalization within30 d of a preceding hospitalization.
14	Over the past 12 mo, the child had a minimum of 1 unplanned hospitalization to the ICU, independent of the length of stay on this unit.
15	Over the past 12 mo, the child had a minimum of 6 cumulative days in the ICU, independent of the number of ICU admissions.
16	The child experienced an ICU readmission within 30 d of a previous ICU admission.
**Family needs**	
17	To care for the medical needs of their child, the caregiver(s) are experiencing an impactful amount of planned or unplanned work disruption.
18	Currently, the caregiver(s) lack the financial resources to pay necessary expenses at the end of the month causing difficulties in providing the necessary care for the child.
19	Over the past 12 mo, the caregiver(s) are incurring an impactful amount (% of the family income) of expenses for the child's medical care beyond what is expected from raising a child without medical issues.
20	Currently, the child lacks medical insurance to cover basic medical needs.
21	Over the past 12 mo, there is a minimum average of 10 h/wk of caregiver time spent on the direct medical care of the child or on care related to ADLs beyond what is age-appropriate need. Note: ADLs include eating, bathing, dressing, toileting, transferring, and maintaining continence.

22	Currently, the caregiver(s) are receiving inadequate amount of respite care at home relative to their self-reported needs.
23	Because of the time and effort to care for the child's higher needs, the family is currently having difficulty performing their daily family activities. Note: Examples of these activities include family outings, home family time, and routine care of the other children.
24	Currently, there are impactful problems in the family relationships, such as communication difficulties or family conflicts, that are resulting from the demands of caring for a child with medical issues.
25	The child is experiencing or witnessing impactful disruption in their social life. Note: This social disruption may include, but is not limited to, caregiver substance use, domestic violence, housing insecurity, food insecurity, neglect of the child, or an active child welfare case.
26	Currently, the primary caregiver(s) are isolated from community or family support which is either negatively impacting the care of the child OR is a result of the care needs of the child.
27	The primary caregiver(s) have developed, or had a worsening of, a physical, psychological, or cognitive ailment since assuming the care of the child.
28	Over the past school year, the child is experiencing a minimum average of 6 d/mo of unplanned school absences due to complications of their medical issues, assuming the child is regularly attending school.
29	Over the past 12 mo, the child has missed an impactful number of scheduled, medically necessary appointments including, but not limited to, preventative or well-child care visits.
30	Currently, the child is failing to receive management that is considered medically necessary and/or is part of the parent's care goals for the child. Note: Management may be considered medication, therapies, or services.
31	Over the past 12 mo, caregivers are having difficulty providing recommended care because the chronic care routines for the child are not stable and frequently changing.
32	Currently, the care of the child is impacted by inadequate care coordination.
33	Over the past 12 mo, there was a minimum average of 5 h/wk of caregiver time spent on care coordination for the child.
34	Currently, the child is experiencing complications resulting from unmet care needs.
35	Currently, the primary caregiver(s) have suboptimal health literacy and numeracy relative to that needed to care for the child.
36	The caregiver(s) are having difficulty accessing and delivering care to the child with medical issues because they are not proficient in the predominant language of their current community.
37	Currently, the caregiver(s) are having difficulty accessing care because the child lives in a remote or rural setting.
38	Currently, the caregiver(s) lack access to reliable, specialized transportation necessary for the child's care.
39	Currently, the child is at risk for rapid medical decompensation or has had frequent life-threatening events at home.

ADL, activities of daily living; VBSF, vital body structures or function.

Adapted from Millar K, Rodd C, Rempel G, Cohen E, Sibley KM, Garland Al. The Clinical Definition of Children With Medical Complexity: A Modified Delphi Study. Pediatrics. 2024;153:e2023064556.
